# Multilocus dataset reveals demographic histories of two peat mosses in Europe

**DOI:** 10.1186/1471-2148-7-144

**Published:** 2007-08-22

**Authors:** Péter Szövényi, Zsófia Hock, Jakob J Schneller, Zoltán Tóth

**Affiliations:** 1 Institute of Systematic Botany, University of Zurich, Zurich, 8008, Zollikerstrasse 107, Switzerland; 2 Department of Plant Taxonomy and Ecology, Eötvös Loránd University, Budapest, 1117, Pázmány Péter sétány 1/C, Hungary

## Abstract

**Background:**

Revealing the past and present demographic history of populations is of high importance to evaluate the conservation status of species. Demographic data can be obtained by direct monitoring or by analysing data of historical and recent collections. Although these methods provide the most detailed information they are very time consuming. Another alternative way is to make use of the information accumulated in the species' DNA over its history. Recent development of the coalescent theory makes it possible to reconstruct the demographic history of species using nucleotide polymorphism data. To separate the effect of natural selection and demography, multilocus analysis is needed because these two forces can produce similar patterns of polymorphisms. In this study we investigated the amount and pattern of sequence variability of a Europe wide sample set of two peat moss species (*Sphagnum fimbriatum *and *S. squarrosum*) with similar distributions and mating systems but presumably contrasting historical demographies using 3 regions of the nuclear genome (appr. 3000 bps). We aimed to draw inferences concerning demographic, and phylogeographic histories of the species.

**Results:**

All three nuclear regions supported the presence of an Atlantic and Non-Atlantic clade of *S. fimbriatum *suggesting glacial survival of the species along the Atlantic coast of Europe. Contrarily, *S. squarrosum *haplotypes showed three clades but no geographic structure at all. Maximum likelihood, mismatch and Bayesian analyses supported a severe historical bottleneck and a relatively recent demographic expansion of the Non-Atlantic clade of *S. fimbriatum*, whereas size of *S. squarrosum *populations has probably decreased in the past. Species wide molecular diversity of the two species was nearly the same with an excess of replacement mutations in *S. fimbriatum*. Similar levels of molecular diversity, contrasting phylogeographic patterns and excess of replacement mutations in *S. fimbriatum *compared to *S. squarrosum *mirror unexpected differences in the demography and population history of the species.

**Conclusion:**

This study represents the first detailed European wide phylodemographic investigation on bryophytes and shows how pattern of nucleotide polymorphism can reveal unexpected differences in the population history of haploid plants with seemingly similar characteristics.

## Background

Geographic distribution of species is continuously shaped by several extrinsic factors causing repeated historical range and demographic expansions/contractions [1,2]. Using historical observations, short-term range expansions and contractions, together with associated demographic changes, have been documented in several, mostly invasive species in Europe and also world-wide [3-6]. From a conservation biological point of view, it is of high importance to reconstruct the historical demography of populations, which can help to work out conservation strategies. In addition, correlations between demographic changes and climatic oscillations may be useful to predict species responses to the accelerated climate change [7]. If a series of temporarily spaced data is available, demographic changes over several decades are relatively easy to reconstruct. However, comparison of historical and recent observations is challenging, among others because of the difficulties emerging when comparing data gathered by different sampling strategies [8].

Methods using the current level and distribution of genetic variability to estimate the historical demography of populations provide an additional tool to test demographic hypotheses suggested by simple historical observations [9-12]. Historical demography considerably influences both qualitative and quantitative properties of sequence-level polymorphisms at neutrally evolving sites [13,14]. Consequently, based on the current pattern of sequence-level polymorphisms, it is possible to distinguish among populations, which currently underwent a demographic expansion and those, which are in a declining phase. Populations after a demographic expansion are described by rapid coalescent events following long branches on the coalescent tree [14]. In the mismatch distribution, long branches lead to an excess of low frequency polymorphisms [15,16].

Unfortunately, selective forces might lead to the same pattern of molecular variation as would be expected in an expanding population. Investigating noncoding, neutrally evolving sequence portions does not overcome the problem of selection because they might be influenced by hitchhiking effects of linked loci [17]. Although selective forces and demography might produce the same pattern of polymorphism, selection affects the genome locally, whereas demography extends its effect on the whole genome. Consequently, investigating multiple, unlinked loci can help to separate the influence of selection and demography on the pattern of nucleotide polymorphism [18,19].

In the present study, we aimed to reconstruct the demographic histories of two peat moss species based on nucleotide polymorphism data. Peat mosses are an ancient plant group with a worldwide distribution [20] and represent a model system for population genetic research in bryophytes [21-24]. In addition, phylogenetically closely related species pairs with similar distribution patterns and life history characteristics provide an outstanding opportunity to test the influence of species-specific demography on nucleotide polymorphism.

To concentrate on the effect of demography and minimize the influence of other possible factors on the pattern and amount of sequence-level variability, two phylogenetically closely related species with similar distributions, ecological requirements and mating systems have been selected. Both species are monoecious (produce antheridia and archegonia on the same gametophore) and distributed from the Iberian Peninsula to Svalbard across Europe. They frequently occur together at the edge of mires under *Alnus *or *Salix *stands and can tolerate higher nutrient concentrations [20,25]. Although they share similar characteristics, recent investigations show that *S. fimbriatum *is more successful in colonizing open soil surfaces and produces more rapidly sporophytes after establishment than *S. squarrosum *[26]. In addition, analyses of past and current distributions of the species show that *S. fimbriatum *is presumably experiencing a rapid expansion at least in some parts of Europe, whereas *S. squarrosum *shows no evidence of a recent population demographic change [27].

Previous investigations on a limited number of accessions using chloroplast sequences, suggested contrasting phylogeographic patterns in the monoecious *S. fimbriatum *and *S. squarrosum *in Europe [28]. Although chloroplast markers allowed drawing of phylogeographic hypotheses, very low resolution of the dataset hampered any further statistical inference. Moreover, the chloroplast genome consists of completely linked loci and thus may be strongly influenced by selective forces. Consequently, it is impossible to separate the effect of demography and natural selection on nucleotide polymorphisms using only chloroplast sequence data.

Therefore, this study aims to investigate the historical demography of the two species in detail using more detailed sampling, and sequence data of three unlinked nuclear regions covering *appr*. 3000 bps of the genome. We intended to differentiate between selective and demographic forces and were particularly interested in the following questions: 1. Does phylogeographic structure depend on the loci investigated? 2. Does multilocus analysis support assumed population expansion of *S. fimbriatum*? 3. What kind of demographic histories could lead to the current amount and pattern of molecular variability?

## Results

### Geographic distribution of haplotypes

**Figure 1:**
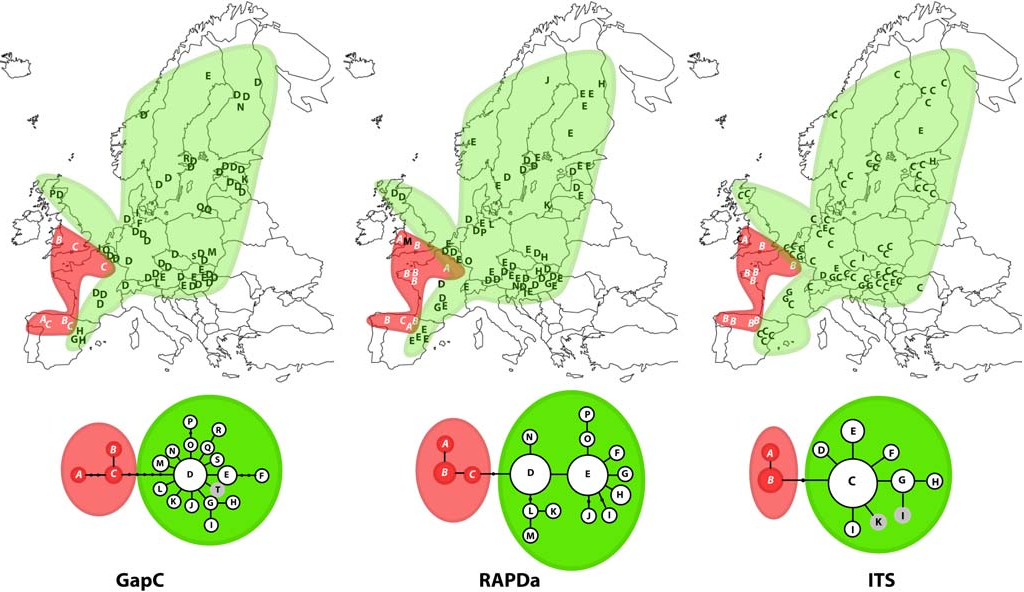
**Geographic distribution of *S. fimbriatum *haplotypes and the corresponding maximum parsimony networks**. In each haplotype network, letters in circles represent haplotypes found. Haplotypes found only in America are shown in circles with gray background. Missing haplotypes are marked with black dots and lines connecting haplotypes denote one mutational change. The Atlantic and Non-Atlantic clades are shown within red and green ellipses respectively.

In *S. fimbriatum*, the GapC and the RAPDa regions resolved the highest number of haplotypes (19 and 16 respectively), whereas the ITS region had much less resolution (9 haplotypes). All three regions supported a well-defined split of haplotypes into two groups (Figure 1). One lineage, further referred to as "Atlantic clade" occurs along the Atlantic coast of Spain, France and southern part of Britain. The rest of the accessions grouped into a clade extending from Southern France to Scandinavia ("Non-Atlantic clade") with one or two frequent and several rare haplotypes. In contrast to the ITS and RAPDa regions, the GapC gene gives further interpretable resolution within the Non-Atlantic clade. Plants from eastern part of Spain (haplotypes G and H, Figure 1) form a separate, geographically well-delimited clade, also accessions from Austria and Hungary (haplotype E, except one occurrence in Scandinavia, Figure 1) show some geographic affinity.

**Figure 2:**
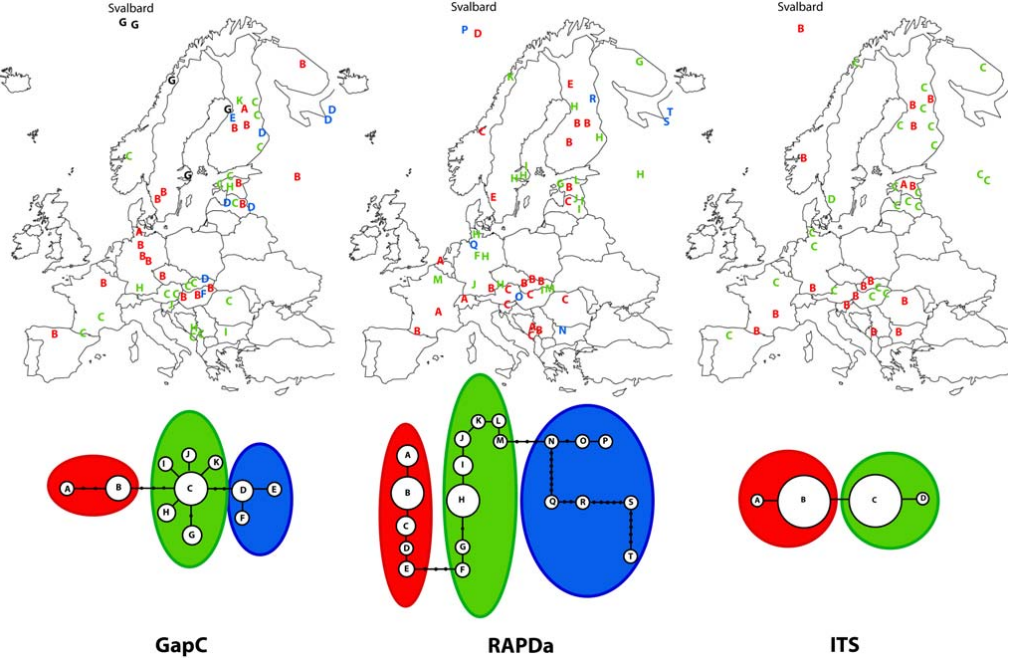
**Geographic distribution of *S. squarrosum *haplotypes and the corresponding maximum parsimony networks**. In each haplotype network, letters in circles represent haplotypes found. Missing haplotypes are marked with black dots and lines connecting haplotypes denote one mutational change. Well-differentiated clades are shown within red, green and blue ellipses. Letters on the map have the colour of the corresponding genetic clade.

In *S. squarrosum*, all three nuclear markers provided similar distributions of haplotypes, however, markers differed much more in their resolution than in *S. fimbriatum *(Figure 2). The ITS region contained only two singleton polymorphisms and one indel, which divided the accessions into two haplotype groups with similar numbers of accessions. The GapC gene resolved 11 haplotypes, whereas in the RAPDa region no substitutions were found except two informative indels. In spite of the lack of substitutions, the RAPDa region contained a complete dinucleotide repeat, which showed considerable variability and resolved 20 haplotypes. The dinucleotide repeat was also present in *S. fimbriatum *sequences but showed no variability. In contrast to *S. fimbriatum*, *S. squarrosum *haplotypes show no clear geographic affinity. One of the ITS types tends to be more frequent in the south than in the north, but with a considerable admixture. RAPDa haplotypes show no clear geographic pattern either. Haplotypes of the GapC gene are also widely distributed and only two of them show any geographic grouping (D, G Figure 2).

### Molecular polymorphism, tests of neutrality and recombination

Estimates of θ were lower in *S. squarrosum *for the ITS region but were similar for the GapC gene in both species (Table 1). The GapC gene of *S. fimbriatum *showed high diversity at synonymous sites whereas in *S. squarrosum *all mutations were found in noncoding regions.

**Table 1:** Nucleotide polymorphisms of the regions investigated. Number of sequences, aligned length, nucleotide diversity estimates and neutrality tests of the nuclear regions investigated. Tajima's D was calculated taking nucleotide substitutions or nucleotide substitutions and gaps into account.

	N	Aligned lenght in bps	θ_w total_	θ_π total_	θ_w syn_	θ_w nonsyn_	θ_w sil_	Tajima's D (with gaps/without gaps)			Fay and Wu's H
	
								Atlantic clade	Non-Atlantic clade	Total data set	Total data set
									
***S. fimbriatum***											
RAPDa	80	993	0.00103	0.00120	-	-	-	-0.6909/-1.1117	-1.7448*/0.4741	-1.3992*/0.3600	0.4044
ITS	90	614	0.00164	0.00077	-	-	-	-1.1117/-1.1117	-1.7460*/-1.6393*	-1.5950*/-1.1427	0.4155
GapC	76	1566	0.00236	0.00101	0.01124	0.00186	0.00267	-0.8764/-1.3272	-2.2321*/-1.9777*	-1.9074*/-1.8090*	-3.2821
***S. squarrosum***											
RAPDa	57	1033	-	-	-	-	-	-	-	-	-
ITS	60	668	0.00033	0.00005	-	-	-	-	-	-0.2501/-1.0857	0.0655
GapC	50	1561	0.00201	0.00202	0.00000	0.00000	0.00201	-	-	-0.4287/0.0279	-1.2114

θ_w total_: Watterson's estimator calculated using all accessions; θ_π total_: Tajima's estimator calculated using all accessions; syn: synonymous sites; nonsyn: nonsynonymous sites; sil: silent sites; * p ≤ 0.05.

The RAPDa region has been excluded from the multilocus HKA test because it showed no point mutations in *S. squarrosum *and only the microsatellite repeat was variable. No significant deviation from the neutral model was detected using the multilocus HKA test (p = 0.48). Despite of the excess of non-synonymous substitutions in *S. fimbriatum *compared to *S. squarrosum *(Table 1), the McDonald-Kreitman test (GapC gene) was not significant either (p = 0.50). In *S. squarrosum*, Tajima's D and Fay and Wu's H statistics were never significantly different from the neutral expectations.

Using all European accessions of *S. fimbriatum*, Tajima's D was significant for all three nuclear regions investigated when including gaps (Table 1). However, it turned out to be non-significant when excluding gaps, except for the GapC gene. Fay and Wu's H was never significant. Analysing accessions of the Non-Atlantic clade of *S. fimbriatum*, Tajima's D was significantly negative except the RAPDa region. The latter showed a significant negative value as well when gaps were included. The Atlantic clade showed no significant deviations from neutrality.

No signs of recombination were found in RAPDa and ITS data sets of both species. GapC sequences of *S. fimbriatum *showed one incompatible pair and an R_m _value of 1. The two accessions causing the incompatibility were removed from the data set in further analyses. No signs of recombination were found in GapC sequences of *S. squarrosum*.

### Population growth

Without gaps, Fu's F_s _statistic was only significant in the case of the GapC gene including all European accessions of *S. fimbriatum *(Table 2). In contrast, when gaps were used in the calculations, it turned out to be significantly different from a constant population in all three regions investigated. Analysing only the Non-Atlantic group of accessions, test statistic was significantly negative in all three genomic regions when including gaps. None of these statistics were significant using European accessions of *S. squarrosum *and accessions of the Atlantic clade of *S. fimbriatum*.

**Table 2:** Test of population growth using Fu's F_s _statistic. Calculations were done including only nucleotide substitutions or nucleotide substitutions and gaps in the analysis.

	Fu's F_s _(with gaps/without gaps)		
	Atlantic	Non-Atlantic	Total data set
	
*S. fimbriatum*			
RAPDa	-0.5938/-0.3393	-8.4558*/0.7145	-8.0260*/0.7850
ITS	-0.3393/nc	-7.1366*/-5.2437*	-6.3052*/-1.7310
GapC	0.5410/0.8564	-15.4390*/-11.5356*	-10.7240*/-7.4648*
*S. squarrosum*			
RAPDa	-	-	-
ITS	-	-	-0.4350/-1.7750
GapC	-	-	-0.6240/1.2670

* p ≤ 0.05

Due to low substitutional variability, ITS and RAPDa regions of both species had to be excluded from the maximum likelihood estimation of exponential growth rate and historical theta. In *S. fimbriatum *estimation was made separately for all European accessions and for the Non-Atlantic clade. Analysis using all European accessions of *S. fimbriatum *showed about 4 times higher growth rates than in *S. squarrosum*, however, values were not significantly different from a shrinking, stable or expanding population (Table 3). Historical theta values were nearly the same for both species. Estimation provided two orders of magnitude higher growth rates and about one order of magnitude higher theta estimates for the Non-Atlantic clade of *S. fimbriatum *compared to *S. squarrosum*. Growth rate of *S. fimbriatum *in the Non-Atlantic clade was significantly different from zero, whereas that of *S. squarrosum *might come from a declining, expanding or stable population as well. Although likelihood surfaces were relatively flat, growth rate values of the two species turned out to be significantly different taking approximate confidence intervals into account.

**Table 3:** Test of population growth using mismatch distribution analysis. Analysis was conducted using all European accessions of both species. Note that estimations are given only for the GapC gene.

Mismatch analysis							
	Pure demographic expansion				Spatial expansion		
		
	p	Tau	θ_0_	θ_1_	p	Tau	θ_0_
		
*S. fimbriatum*	0.026	-	-	-	0.710	0.325 (0.210–4.710)	0.782 (0.000–1.652)
*S. squarrosum*	0.143	4.976 (1.860–9.362)	0.000 (0.000–1.813)	6.509 (2.843–88.003)	0.257	4.195 (1.678–7.446)	0.092 (0.000–1.995)

p: significance of fit (observed vs. expected distributions); Tau: scaled time elapsed since the expansion; θ_0_: theta of the population before expansion; θ_1_: theta of the population after expansion; 90% confidence intervals are given in brackets.

In the mismatch distribution analysis, only the GapC sequences were used because of the low variability of the RAPDa and ITS regions (Table 3 and 4). The sudden demographic and the spatial expansion model both fit mismatch data of *S. squarrosum*. However, the fit was better using the spatial expansion model. The mismatch distribution of all European accessions of *S. fimbriatum *was significantly different from the pure demographic expansion model, but matched the spatial expansion model. Tau was at least 4 times larger in *S. squarrosum *than in *S. fimbriatum*. Theta estimated by the spatial expansion model was greater in *S. fimbriatum*, but this difference was not statistically significant. Estimation of parameters of sudden population growth or population expansion models failed for the Non-Atlantic group of *S. fimbriatum*, because the non-linear least squares algorithm failed to converge in ARLEQUIN.

**Table 4:** Test of population growth using maximum likelihood estimation. Analysis was conducted using all European accessions of both species. Note that estimations are given only for the GapC gene.

Maximum likelihood estimates				
	Non-Atlantic clade		Europe	
		
	θ	g	θ	g
		
*S. fimbriatum*	0.01218 (0.00393–0.06008)	15000.00 (7831.62–32513.97)	0.00377 (0.00176–0.00678)	857.04 (-622.83–5891.93)
*S. squarrosum*	-	-	0.00242 (0.00113–0.00563)	233.58 (-1244.40–2401.97)

θ: maximum likelihood estimator of the neutral mutation parameter; g: the exponential growth parameter; 95% percentile intervals of values around the most likely estimates are shown in brackets.

### Fitting the data to the IM model

**Figure 3:**
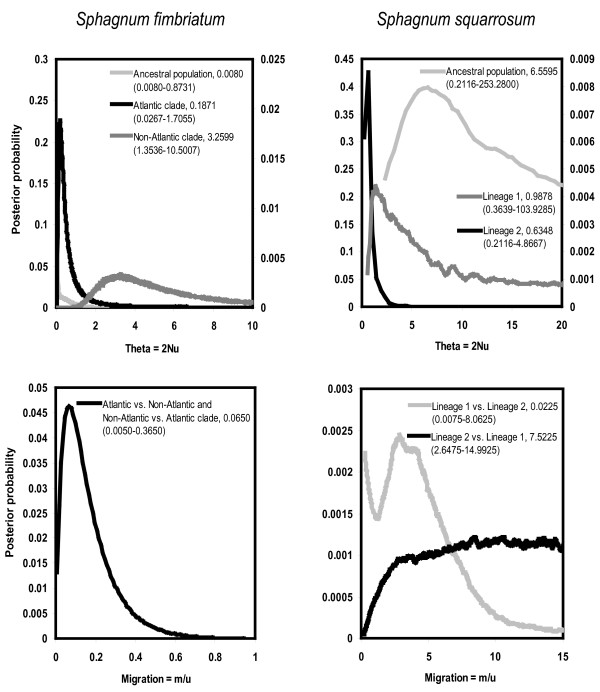
**Posterior probability density functions of the parameters estimated using the Isolation with Migration model**. In the figures point estimates of each parameter and the 90% highest posterior density intervals (in brackets) are given for each parameter. Note that parameter estimates are scaled with mutation rates. N: effective population size, m: rate of migration per gene per generation, u: mutation rate per gene per generation.

The provided population genetic parameter estimates of the IM model are all scaled with the mutation rate. In the absence of reliable mutation rate estimates of the regions results were interpreted in a relative way (Figure 3). Current theta values of the Atlantic and Non-Atlantic clades of *S. fimbriatum *showed considerably different distributions and point estimates. The Atlantic clade had lower theta than the Non-Atlantic clade and theta of the ancestral population was much lower than both of them. Estimate of theta for the Non-Atlantic clade was flat and converged but did not reach zero even after considerable extension of the prior. Scaled migration parameters were all very close to zero. Point estimate of the splitting parameter was high.

In contrast to *S. fimbriatum*, current thetas in *S. squarrosum *almost gave the same point estimates. Point estimate of the ancestral neutral mutation parameter was an order of magnitude higher than that of the current populations, but the posterior distribution was relatively flat. It converged to zero at higher theta values but did not reach zero even after extending the prior of theta considerably. Scaled migration parameters showed non zero values but were flat. Posterior probability distribution of the splitting parameter was flat and uninformative (not shown).

## Discussion

### Phylogeographic structure

Results of the present analysis confirm the divergence of an Atlantic and a Non-Atlantic lineage in *S. fimbriatum *and almost no geographic population structure in *S. squarrosum *[28]. Moreover, our current results evidently support a genome-wide historical event, which influenced all regions in the same way regardless of genomic position.

Since there is no reliable calibration point in the history of the genus, it is not possible to date the split in *S. fimbriatum*. In absence of dating, at least two hypotheses are plausible. The first one assumes that Quaternary cold periods were long enough to allow accumulation of the observed number of mutations among the Atlantic and Non-Atlantic groups of populations. In addition, it presumes that current geographic position of populations roughly correspond to that of the historical ones. The Atlantic group might have survived along the Atlantic coast of Spain, France and South England, but location of refugia of the Non-Atlantic group remains ambiguous. Results suggest that Eastern Iberian populations represent recent colonization events. Using reconstructions of Gajewski *et al*. [29] based on peat moss spores we hypothesize survival of the Non-Atlantic lineage along the border of Poland and Slovakia where extensive *Sphagnum *dominated habitats occurred in about 19 ky before present.

The second hypothesis assumes intercontinental migration between Europe and America. North American and European populations of several *Sphagnum *species appear to be closely related [30-33]. Among others, long-range dispersal as a plausible explanation for the observed pattern has been suggested [26,34,35]. Assuming effective intercontinental migration, colonization from a divergent American population might account for the difference between the two lineages. To test this hypothesis we analysed additional samples from different parts of North America. Most of the North American accessions either shared haplotypes with the Non-Atlantic group or differed by only one substitution and represented derived tip haplotypes. Therefore recent colonization from America does not account for the divergence of the Atlantic and Non-Atlantic clades.

In addition, several issues indicate that the Quaternary period has played an important role in the observed genetic structure of the species. First, the patterns detected in this study were very similar to those found in other bryophyte species [36-38] and seed plants [39,40]. The second reason comes from fossil data and climatic reconstructions. Occurrence of relic *Sphagnum *species [20], fossil remains of peat moss spores or leaves [41,42] and climatic reconstructions also support survival of populations along the Atlantic coast of Europe [29,43]. Consequently, our results and the above-mentioned observations support the separation of the Atlantic and Non-Atlantic lineages of *S. fimbriatum *by Quaternary glacial cycles and reject recent colonization from another continent as the cause of the split.

### Population demography

Selection influences each gene differently, whereas demographic processes affect the individual as a whole and thus influence all parts of the genome uniformly regardless of genomic position [18]. Hence, in an expanding population an excess of low frequency variants is expected for all loci. This leads to negative Tajima's D and Fu's F_s _statistics [44,45]. These statistics all analyse the total or relative number of singleton mutations compared to the neutral coalescent. Their power depends on the total number of segregating sites in the data set and the time elapsed since the demographic event [45,46]. Therefore it is not surprising that some of the statistics were not significant when excluding gaps as segregating sites from the analyses. This shows that substitution rates are relatively low compared to the time elapsed since the demographic event. Increasing the mutation rate with including gaps as mutations in the calculations improves power of the tests and extends the time frame in which those are applicable. Using these assumptions, all test statistics for all regions of *S. fimbriatum *were significantly different from the neutral expectations, whereas none of them provided significant deviations in *S. squarrosum*. This confirms a general excess of low frequency variants, in *S. fimbriatum *especially in the Non-Atlantic clade. Since the regions used are unlinked, we favour a population demographic expansion against a genome wide selection hypothesis in *S. fimbriatum*. Sequencing errors are negligible because both species were analysed using the same protocols and polymorphisms have been rechecked on the original chromatograms.

Mismatch distribution analysis revealed further details about the demographic history of the two species. Mismatch distribution of spatial expansion only slightly differs from that of pure demographic expansion, the latter showing a higher frequency of identical sequences [47]. In case of *S. fimbriatum*, data support the spatial expansion model, which is in concordance with the hypotheses about rapid spatial expansion of the species after the Quaternary glaciations. Data from *S. squarrosum *fit both scenarios, however, considerably better match the latter. This suggests that *S. squarrosum *probably went through spatial expansion as well. Tau value of *S. squarrosum *was approximately an order of magnitude higher than that of *S. fimbriatum*, although they were not significantly different. This suggests that expansion of *S. fimbriatum *is more recent than that of *S. squarrosum*. Assuming that *S. fimbriatum *expanded immediately after the Last Glacial Maximum (LGM), the estimate for *S. squarrosum *suggests expansion related to earlier geological or climatic events than the LGM. However, results of mismatch analyses need to be interpreted with caution. These analyses do not use genealogical information included in the data and testing the fit between observed and expected distributions and estimating approximate confidence intervals represent a complex problem as well. Estimation and confidence intervals of tau and θ are skewed and biased, especially in data sets with low resolution [48]. Maximum likelihood (LAMARC) and Bayesian analyses (IM) also supported rapid population expansion in *S. fimbriatum*. Results of both analyses showed no significant evidence for population growth in *S. squarrosum*. The IM analysis even supported a population decline since the separation of the two lineages of *S. squarrosum*.

Information on the nucleotide polymorphism of three independent regions gives the opportunity to reconstruct the historical demography of the two species in details. Populations of *S. fimbriatum *and *S. squarrosum *have been separated by the Quaternary glaciations, which led to the differentiation of two and three genetically well diverged European lineages, respectively. Fixed replacement mutations among the Atlantic and Non-Atlantic groups of *S. fimbriatum *indicate that both lineages went through a severe bottleneck, which led to the fixation of slightly deleterious mutations. It is worth noting that populations of the Atlantic group retained relatively divergent haplotypes (Figure 1) despite of their scattered occurrence and small extent (occurrences are usually not larger than 5 × 5 m). Hence, these populations very likely represent the leftovers of a formerly more widely distributed genetic lineage. Fitting the IM model to the data set suggests that a very high proportion of the ancient population of *S. fimbriatum *founded the Atlantic clade and the size of the ancient population was very small. These results show that the Atlantic clade is very likely a direct descendant of the ancestral population, which retained ancestral haplotypes and went through a severe bottleneck. IM and maximum likelihood estimations show that the Quaternary glaciations shaped population size of the two species differently. Ancient population size of *S. squarrosum *is considerably larger than that of *S. fimbriatum *indicating a more severe bottleneck in the latter. Larger effective population size of the ancient population of *S. squarrosum *also explains the lack of replacement mutations in coding regions as well as the lack of geographic structure of haplotypes.

### Pattern and amount of molecular variability

Overall diversity values for the ITS region were usually an order of magnitude higher in *S. fimbriatum *than in *S. squarrosum*, however all values were very low. Moreover, in *S. fimbriatum*, a considerable portion of the diversity was due to the genetic differentiation between Atlantic and Non-Atlantic clades. In contrast, GapC sequences of the species showed nearly the same nucleotide diversity values. This is an unexpected result because the pioneer characteristic of *S. fimbriatum *(Sundberg *et al*. 2006), even when combined with a parallel reduction of the historical population size of *S. squarrosum*, implies lower level of molecular polymorphism in the first species. Peat moss populations are likely to function as a metapopulation [49,50]. Theory shows that species-wide genetic diversity is rapidly lost in metapopulations of pioneer species if among population migration rates are lower than extinction/recolonization rates [51,52]. Although pioneer characteristic reduces the level of molecular diversity, in range expansion, due to the phenomenon of surfing mutations, molecular diversity is increased by a higher proportion of retained neutral mutations [53]. Consequently, it seems likely that range expansion contributed significantly to the molecular variability of *S. fimbriatum*.

Although the magnitude of molecular polymorphism was similar in both species, its pattern differed considerably showing an excess of polymorphism in coding regions of *S. fimbriatum *compared to *S. squarrosum*. Recent increase of effective population size results in a significant excess of slightly deleterious replacement mutations [54]. Weakened selection due to reduced effective population size can lead to the accumulation of slightly deleterious mutations as well and thus to elevated nucleotide diversity in selfing plants [55]. The McDonald-Kreitman test showed no significant deviations from neutral expectations for the GapC gene, however, exons of this gene contained replacement mutations in *S. fimbriatum *whereas in *S. squarrosum *all mutations were restricted to noncoding regions. Replacement mutations in *S. fimbriatum *(Lys-Arg, Ile-Val, Lys-Gln) caused no change in hydrophoby and almost no change in pI values and thus are likely to be only slightly deleterious. Consequently, elevated number of replacement mutations in *S. fimbriatum *also supports a rapid population demographic expansion in Europe.

Both species are easily dispersed by spores to longer distances [26,27], however, nuclear markers resolved distinct Atlantic and Non-Atlantic clades only in *S. fimbriatum*. All markers indicate either restricted migration or establishment potential measured on the time scale of the markers' mutation rates. *S. fimbriatum *produces considerable amounts of spores, which can germinate on a wider range of substrates (especially on substrates with low phosphate availability) compared to other *Sphagnum *species [26,56]. Based on these characteristics of the species, the Non-Atlantic clade of *S. fimbriatum *might have rapidly recolonized available soil surfaces after the last glaciation. In absence of further space, the Atlantic clade might have been unable to follow this lineage and remained restricted to the Atlantic coast of Europe. Similar patterns of leading edge colonization have been observed in several tree species in Europe [57,58].

Overall nucleotide diversity of the three regions was low in both species compared to estimations in seed plants [reviewed in [18]], which might be a general trend in bryophytes. Due to the haploid gametophores, no sheltering of recessive alleles exists in bryophytes and the haploid genome of each individual is directly exposed to selection [30,59]. This, coupled with the presence of deleterious mutations, considerably reduces the amount of genetic diversity. Mutation rate also influences genetic variability. Selection against high mutation rates should exist in bryophytes; otherwise the presence of slightly deleterious mutations will lead to a severe mutational load [60]. Our results show that slightly deleterious mutations are present but are removed very efficiently. The theory of selection directly acting on the gametophores and low mutation rates are supported by the data set presented here. Assuming the same mutation rates in both species, selection is so efficient, that no replacement mutations were found in the GapC gene of *S. squarrosum *at all. Interestingly, in a previous study McDaniel and Shaw [61] investigated the level of nucleotide polymorphism in the moss *Ceratodon purpureus *which turned out to be an order of magnitude higher than in our species. As this investigation relied on a worldwide sample and used multi-copy genes, high level of polymorphism may reflect the complex interplay of various forces, such as evolution of gene families, selective sweeps and phylogeographic history. Hence, the two studies are hardly comparable and further investigations on the extent of population level sequence polymorphism are needed to test theoretical predictions. In contrast to our results, high levels of isozyme diversity were found within populations of several bryophytes [30,60]. This has partly been interpreted as a result of local adaptation [61]. Although local adaptation might be important in several species, our results do not support this hypothesis, since only one of the three replacement mutations in the GapC gene resulted in charge change. Hence these mutations probably do not influence enzyme functions significantly.

## Conclusion

Analysing nucleotide polymorphisms of multiple loci combined with the application of the coalescent theory provides an effective way to draw inferences about historical demography of species. Furthermore, investigating polymorphisms of several independent loci makes it possible to separate the effects of selective and demographic forces. Our study evidently shows that species with similar ecology, mating systems and current distributions may have very different demographic and phylogeographic histories. Such cryptic differences are probably frequent among bryophytes and must be taken into account when assessing the vulnerability of species and planning conservation strategies. Due to their haploid gametophores, bryophytes are especially appropriate for studies on sequence level polymorphisms. Future research using nucleotide polymorphism data will certainly discover undetected demographic, phylogeographic and evolutionary processes in this group of haploid plants.

## Methods

### Sampling and DNA extraction

*S. fimbriatum *and *S. squarrosum *plants were collected by the authors, provided by colleagues or obtained from herbaria [see Additional file 1]. Plants were dried and stored in silica gel at 4°C. DNA was extracted using the DNAeasy plant mini kit (Qiagen, Switzerland) following the manufacturers protocol. Three regions of the nuclear genome were used for sequencing. The region spanning the 5th–9'th exons of the GapC (glyceraldehyde-3-phosphate-dehydrogenase C) gene (appr. 1500 bps) was amplified using protocols and primers described in Szövényi *et al*. [64]. A genomic region with unknown function [RAPDa sensu [65]] was also used. New primers were designed to improve amplification and sequencing of the fragment. As a third locus the ITS1-5.8S-ITS2 region (hereafter ITS) were amplified and sequenced. Yield of PCR reactions were improved by applying a semi-nested PCR, where 1 μl of the 10 × diluted first round product was used as template in the second reaction [see Additional file 2]. The PCR reaction contained the same amount of components as described in Szövényi *et al*. [64] except that final DMSO concentration was set to 1% v/v in the PCR mastermix to amplify the ITS region. PCR reactions were run on Biometra (T1 or Tgradient, Whatman Biometra) and Techne ptc 412 (Barloworld Scientific Ltd.) thermocyclers. The PCR program was the following: 94°C denaturation for 4 min, then 94°C for 1 min, different annealing temperatures for 1 min and 72°C extension for 1–1.5 min [see Additional file 2] with 35 cycles with a final extension step at 72°C for 7 min. The products were checked on 0.8% agarose gels and cleaned using the GFX PCR and gel band purification kit (Amersham Biosciences, Switzerland). Approximately 10 ng product was sequenced in 10 μl cycle sequencing reaction with the Big dye v3.1 in an ABI prism 3100 (Applied Biosystems) genetic analyzer using either the original and/or different internal PCR primers [see Additional file 2]. Sequences were contiged and corrected if necessary with the Sequencher 4.5 software (Gene Code Corporation).

### Sequence alignment, molecular diversity and recombination event estimates

Sequences were aligned using ClustalW [66], checked by eye and adjusted if needed. All polymorphic sites were rechecked on the original electropherograms and corrected to avoid false base callings. Haplotype networks were reconstructed using the TCS software [67]. To describe the infra specific molecular diversity of the regions Watterson's (θ_w_) [68] and Tajima's (θ_π_) [44] moment estimators of the neutral mutation parameter were calculated. In case of the GapC gene, levels of polymorphism were calculated for synonymous, nonsynonymous and silent sites as well.

All regions were tested for recombination using the four gamete test and the minimum number of recombination events during the history of the sample was calculated [R_m_, [69]]. For several following analyses (cf. below) sequences with incompatible nucleotide pairs were cut into recombination free blocks. All calculations were done using dnaSP v.4.10.4 [70].

### Testing deviation from neutrality

To test whether the sequenced regions evolved under neutral expectations, four different methods were applied. The multilocus HKA test was conducted using the HKA software [71]. 10 000 coalescent simulations were run to generate the null distribution of χ^2 ^values. Neutral evolution of the GapC gene was also tested with the McDonald-Kreitman test [72]. To test the neutraliy of each locus separately, Tajima's D [44] and Fay and Wu's H statistics [73] were calculated for each of them. For each locus the latter two test statistics were calculated considering nucleotide substitutions alone and recoding gaps as well. Gaps were coded as single and independent events. Due to the low variability and non-overlapping occurrence of gaps in the data set, alignment, assessment of homology and subsequent coding of gaps was straightforward.

### Analysis of population growth

As a powerful descriptive statistic of population growth, Fu's F_s _[74], was calculated using frequency distribution of alleles for each region separately. Fu's F_s _statistic compares the number of alleles expected under the neutral coalescent in a constant sized population based on the neutral mutation parameter and the size of the sample. In an expanding population large negative values are expected due to the excess of rare mutations. Populations after a demographic expansion show a star-like phylogeny and a unimodal mismatch distribution [15], where the peak corresponds to the time of the expansion. The method of Schneider and Excoffier [48] implemented in ARLEQUIN 3.0 [75] was used to fit observed and expected mismatch distributions and to estimate parameters of a pure demographic and a spatial expansion model. The two models are expected to produce different mismatch distributions when the number of migrants between populations is not large. Otherwise the two hypotheses can not be distinguished. In this study both models were considered to complement genealogical methods that fail to detect signs of a spatial expansion.

As an alternative, maximum likelihood estimation of the exponential growth rate and historical θ was conducted. The software LAMARC 2.0 [11] was used to get estimates of growth rates for all European accessions of *S. squarrosum *and *S. fimbriatum *and for the Non-Atlantic clade of *S. fimbriatum*. 30 short chains (20 000 steps each) and 2 long chains (200 000 steps) with a sampling interval of 20 steps and a burn in period of 1000 and 10 000 respectively were applied.

The IM software [76,77] was used to fit the data to the Isolation with Migration model (IM). This model assumes two populations connected by migration, which are derived by the splitting of an ancient population. The IM model estimates several parameters simultaneously and therefore describes processes in natural populations in their complexity. Populations were defined based on our a priory knowledge of chloroplast lineages using the parsimony networks of each species [28]. Runs were conducted for the combined dataset including all three nuclear regions sequenced. Initial runs with wide priors were used to delimit the plausible range of priors. Since indels of all three regions were informative, analyses have been conducted both with and without indels coded. Including gaps as additional characters did not change the multilocus parameter estimates but narrowed their confidence intervals, however, only results without coding gaps are shown. The infinite site mutation model of sequence evolution was applied and loci were cut into non-recombining blocks. In case of the RAPDa sequences of *S. squarrosum*, only the microsatellite region was used. Each run was introduced by a 100 000 steps long burn-in period. Multilocus analyses were run using 5–7 parallel chains under a linear heating scheme with a heating value of 0.05 – 0.005. Convergence of parameters and mixing of chains were followed by visual inspection of parameter trend lines and checking of ESS values. Analyses were run until the lowest ESS value reached minimum 200. In all analyses population demographic changes were allowed.

## Authors' contributions

PS and ZH initiated the study, carried out the molecular genetic studies, analysed the data and wrote the manuscript draft. ZT intensively helped in collecting. JJS and ZT supervised the work, contributed to the analyses of the results and to the writing of the paper. All authors read and improved the final manuscript.

## Supplementary Material

Additional file 1 Collection data and GenBank numbers of accessions analysed. Detailed list of the *Sphagnum fimbriatum *and *S. squarrosum *accessions analysed for the three regions in this study. Letters refer to haplotypes of Figure 1 and 2; na: not analysed. Click here for file

Additional file 2 Primers used in this study. Names of newly designed primers are in bold. 1st and 2nd refer to the first and the second round of a semi nested PCR. In the second round PCR 1 μl 10 × diluted first round product was used as template. Click here for file
