# Supplementary material for: Multilocus dataset reveals demographic histories of two peat mosses in Europe
Source: BMC Evol Biol. 2007 Aug 22;7:144. doi: 10.1186/1471-2148-7-144 (PMC2018716; doi:10.1186/1471-2148-7-144)
Supplement: Additional file 1 — Collection data and GenBank numbers of accessions analysed. Detailed list of the Sphagnum fimbriatum and S. squarrosum accessions analysed for the three regions in this study. Letters refer to haplotypes of Figure 1 and 2; na: not analysed. [file 1471-2148-7-144-S1.pdf]

Additional file 1 Detailed list of the *Sphagnum fimbriatum* and *S. squarrosum* accessions analysed for the three regions in this study. Letters refer to haplotypes of Figure 1 and 2. na: not analysed.

Species	Number of populations	Location	m a.s.l.	Longitude/latitude		Haplotype			GenBank accession number		
						GapC	RAPDa	ITS	GapC	RAPDa	ITS
Sphagnum fimbriatum	1	Scotland, Forest of Alyth	400 m	56° 30' N	03° 20' W	P	D	C	EF184680	EF184821	EF184725
Sphagnum fimbriatum	2	Scotland, Trinafour	300 m	56° 45' N	03° 55' W	D	D	C	EF184710	EF184836	EF184726
Sphagnum fimbriatum	8	Hungary, Alsó-Erdö I	330 m	47° 24' N	16° 33' E	M	D	C	EF184699	EF184834	EF184727
Sphagnum fimbriatum	9	Hungary, Büdöskút-Árpádforrás	435 m	47° 22' N	16° 28' E	D	E	G	EF184673	EF184862	EF184728
Sphagnum fimbriatum	10	Hungary, Büdöskút-Árpádforrás	435 m	47° 22' N	16° 28' E	D	I	C	EF184678	EF184881	EF184729
Sphagnum fimbriatum	14	Norway, Sor-Trondelag	10 m	63° 42' N	08° 46' E	D	E	C	EF184658	EF184839	EF184730
Sphagnum fimbriatum	56	Bohemia, Trebon	-	48° 46' N	14° 42' E	D	na	C	EF184668	-	EF184732
Sphagnum fimbriatum	63	Bohemia, Stare Jezero	-	48° 46' N	14° 42' E	D	D	C	EF184669	EF184879	EF184733
Sphagnum fimbriatum	65	Bohemia, Krkonoše Mts.	1020 m	50° 45' N	15° 33' E	D	na	I	EF184656	-	EF184734
Sphagnum fimbriatum	67	Bohemia, Chlum u trebone	-	49° 27' N	13° 35' E	D	E	C	EF184653	EF184863	EF184735
Sphagnum fimbriatum	69	Hungary, Velencei-tó	-	47° 11' N	18° 33' E	D	D	C	EF184675	EF184838	EF184736
Sphagnum fimbriatum	70	Hungary, Szigetcsép Csupics-sziget	-	47° 15' N	18° 59' E	E	D	C	EF184665	EF184880	EF184737
Sphagnum fimbriatum	72	Hungary, Dorog	150 m	47° 43' N	18° 45' E	E	na	F	EF184659	-	EF184738
Sphagnum fimbriatum	75	Hungary, Monostori-tó	345 m	46° 54' N	17° 35' E	D	D	C	EF184681	EF184878	EF184739
Sphagnum fimbriatum	79	Austria, Elixhausen	560 m	47° 53' N	13° 01' E	L	E	C	EF184671	EF184856	EF184740
Sphagnum fimbriatum	84	Upper Austria, Tarsdorf	480 m	48° 05' N	12° 51' E	D	D	C	EF184677	EF184866	EF184741
Sphagnum fimbriatum	93	Germany, Durchenbergried	430 m	47° 45' N	08° 58' E	D	na	D	EF184713	-	EF184742
Sphagnum fimbriatum	100	Germany, Hüven	10 m	52° 46' N	07° 34' E	D	E	C	EF184682	EF184849	EF184746
Sphagnum fimbriatum	102	Germany, Butterloch	200 m	51° 35' N	10° 18' E	D	na	C	EF184652	-	EF184743
Sphagnum fimbriatum	108	Sweden, Mulön	-	65° 37' N	22° 17' E	D	D	C	EF184712	EF184890	EF184744
Sphagnum fimbriatum	109	Sweden, Bensbyn	-	65° 38' N	22° 13' E	E	J	C	EF184661	EF184891	EF184745
Sphagnum fimbriatum	110	Germany, Neunkirchen	10 m	52° 46' N	08° 44' E	D	D	E	EF184683	EF184894	EF184747
Sphagnum fimbriatum	114	Germany, Hamberger Moor	15 m	53° 16' N	08° 51' E	F	P	C	EF184660	EF184837	EF184748
Sphagnum fimbriatum	118	France, Troncais forest	280 m	49° 52' N	04° 38' E	D	D	G	EF184692	EF184845	EF184749
Sphagnum fimbriatum	120	Spain, Candin, Lamela	850 m	42° 57' N	06° 41' W	A	B	B	EF184648	EF184870	EF184750
Sphagnum fimbriatum	121	Spain, Onate, Sierra de Elguea-Urkilla	1045 m	42° 57' N	02° 24' W	B	B	B	EF184646	EF184847	EF184751
Sphagnum fimbriatum	122	Spain, Orihuela del Tremedal	1675 m	40° 32' N	01° 38' W	na	E	C	-	EF184851	EF184752
Sphagnum fimbriatum	132	Belgium, Nonceveux	100 m	50° 26' N	05° 43' E	O	D	C	EF184679	EF184873	EF184753
Sphagnum fimbriatum	137	Latvia, sample 4	-	56° 40' N	25° 55' W	D	E	C	EF184696	EF184889	EF184754
Sphagnum fimbriatum	138	Latvia, sample 3	-	56° 40' N	25° 55' W	D	D	C	EF184695	EF184882	EF184755

Continued

Species	Number of populations	Location	m a.s.l.	Longitude/latitude	Haplotype			GenBank accession number		
					GapC	RAPDa	ITS	GapC	RAPDa	ITS
Sphagnum fimbriatum	139	Latvia, sample 2	-	56° 37' N 26° 20' E	D	F	C	EF184693	EF184857	EF184756
Sphagnum fimbriatum	145	Germany, Rheinland-Pfalz	120 m	49° 24' N 07° 42' E	D	O	C	EF184698	EF184841	EF184757
Sphagnum fimbriatum	148	Hungary, Templom-tó	250 m	47° 01' N 16° 49' E	E	D	C	EF184663	EF184868	EF184758
Sphagnum fimbriatum	149	Hungary, Fias-tó	250 m	59° 38' N 19° 17' E	na	E	C	-	EF184823	EF184759
Sphagnum fimbriatum	150	Hungary, Füzes-tó	250 m	47° 02' N 16° 48' E	na	na	C	-	-	EF184760
Sphagnum fimbriatum	152	Hungary, Köcse-tó	250 m	47° 01' N 16° 49' E	E	N	G	EF184664	EF184867	EF184761
Sphagnum fimbriatum	153	France, Walleis-Auemberg	-	50° 14' N 03° 41' E	C	A	B	EF184647	EF184892	EF184762
Sphagnum fimbriatum	166	Finland, Ruovesi	-	62° 04' N 24° 21' E	na	E	E	-	EF184827	EF184763
Sphagnum fimbriatum	169	Finland, Ylikiiminki	-	64° 53' N 26° 07' E	D	E	C	EF184694	EF184859	EF184764
Sphagnum fimbriatum	170	Finland, Oulu	-	64° 34' N 25° 33' E	D	E	C	EF184697	EF184858	EF184765
Sphagnum fimbriatum	171	Finland, Kuusamo	-	65° 48' N 29° 50' E	D	H	C	EF184655	EF184822	EF184766
Sphagnum fimbriatum	172	Finland, Piippola	-	64° 13' N 25° 48' E	N	E	C	EF184714	EF184887	EF184767
Sphagnum fimbriatum	173	Belgium, Province of Liège	-	50° 12' N 05° 26' E	D	E	G	EF184674	EF184864	EF184768
Sphagnum fimbriatum	174	Belgium, Province of Liège	300 m	50° 14' N 05° 27' E	D	D	C	EF184657	EF184883	EF184769
Sphagnum fimbriatum	178	Slovakia, Poprad Basin	680 m	49° 03' N 20° 17' E	E	D	C	EF184662	EF184825	EF184770
Sphagnum fimbriatum	234	Sweden, Lilla Idskär (island)	3 m	59° 38' N 19° 17' E	R	D	C	EF184666	EF184874	EF184771
Sphagnum fimbriatum	235	Sweden, Storön (island)	2 m	59° 27' N 19° 30' E	na	D	C	-	EF184869	EF184772
Sphagnum fimbriatum	236	Sweden, Rödskäret (island)	5 m	59° 36' N 19° 27' W	D	E	C	EF184711	EF184860	EF184773
Sphagnum fimbriatum	237	Sweden, Norra Skrakskär (island)	5 m	59° 35' N 19° 20' E	D	D	na	EF184707	EF184884	-
Sphagnum fimbriatum	251	Austria, Kneisselmoor	-	47° 45' N 13° 01' E	E	D	C	EF184667	EF184828	EF184774
Sphagnum fimbriatum	252	Austria, Zehmemoos	-	47° 59' N 12° 55' E	D	D	E	EF184676	EF184875	EF184775
Sphagnum fimbriatum	253	Switzerland, Les Ponts-de-Martel	1000 m	46° 58' N 06° 43' E	D	E	C	EF184670	EF184852	EF184776
Sphagnum fimbriatum	254	Austria, Wengermoor	-	47° 55' N 13° 10' E	D	E	G	EF184691	EF184888	EF184777
Sphagnum fimbriatum	257	Hungary, Nyíres-tó	150 m	47° 49' N 22° 25' E	D	E	C	EF184709	EF184853	EF184778
Sphagnum fimbriatum	258	Hungary, Nyíres-tó	150 m	47° 49' N 22° 25' E	D	E	C	EF184672	EF184854	EF184779
Sphagnum fimbriatum	260	France, locality 2005/2	-	45° 51' N 02° 10' E	D	E	C	EF184708	EF184865	EF184780
Sphagnum fimbriatum	261	Spain, Barranco Larreakorta	700 m	43° 00' N 02° 49' W	C	C	B	EF184650	EF184872	EF184781
Sphagnum fimbriatum	262	Spain, Peña la Gallina	1620 m	40° 32' N 01° 42' W	G	E	C	EF184684	EF184885	EF184782
Sphagnum fimbriatum	266	France, locality 2005/4	-	45° 49' N 01° 59' E	D	D	G	EF184715	EF184896	EF184783
Sphagnum fimbriatum	267	Spain, Los Ojos	1310 m	40° 32' N 01° 38' W	G	E	C	EF184687	EF184833	EF184784
Sphagnum fimbriatum	268	Spain, Los Ojos	1310 m	40° 32' N 01° 38' W	H	E	C	EF184685	EF184861	EF184785

Continued

Species	Number of populations	Location	m a.s.l.	Longitude/latitude	Haplotype			GenBank accession number		
					GapC	RAPDa	ITS	GapC	RAPDa	ITS
Sphagnum fimbriatum	269	Spain, Candin, Lamela	861 m	42° 57' N 06° 41' W	C	B	B	EF184649	EF184871	EF184786
Sphagnum fimbriatum	297	Poland, Babezyna Dolina	-	49° 46' N 19° 03' E	na	na	C	-	-	EF184787
Sphagnum fimbriatum	298	Poland, Silesian lowland	-	51° 06' N 18° 22' E	M	D	C	EF184700	EF184829	EF184788
Sphagnum fimbriatum	299	Poland, Pagory jaworznickie hills	-	50° 07' N 19° 13' E	D	E	C	EF184716	EF184832	EF184789
Sphagnum fimbriatum	300	Hungary, Regéc	300 m	48° 26' N 21° 26' E	na	na	C	-	-	EF184790
Sphagnum fimbriatum	301	Rumania, Hargita	-	47° 43' N 25° 11' E	na	na	C	-	-	EF184791
Sphagnum fimbriatum	302	Poland, Beskid Makowski Mountains	425 m	49° 40' N 19° 45' E	D	na	E	EF184704	-	EF184792
Sphagnum fimbriatum	303	Hungary, Lókosár	-	48° 28' N 20° 30' E	D	G	E	EF184703	EF184830	EF184793
Sphagnum fimbriatum	304	Hungary, Nagymohos	290 m	48° 15' N 20° 13' E	E	D	C	EF184701	EF184886	EF184794
Sphagnum fimbriatum	305	Hungary, Springs of Tegda Valley	300 m	48° 23' N 21° 34' E	na	D	C	-	EF184876	EF184795
Sphagnum fimbriatum	306	Hungary, Kismohos	300 m	48° 15' N 20° 13' E	na	H	C	-	EF184826	EF184796
Sphagnum fimbriatum	307	Poland, Beskid Slaski Mts.	860 m	49° 38' N 19° 09' E	S	H	G	EF184702	EF184824	EF184797
Sphagnum fimbriatum	340	Germany, Spiekeroog (island)	2 m	53° 46' N 07° 41' E	D	D	E	EF184690	EF184877	EF184798
Sphagnum fimbriatum	342	Russia, Rybachy	5 m	55° 09' N 20° 49' E	Q	K	C	EF184720	EF184831	EF184799
Sphagnum fimbriatum	343	Germany, Jagen	12 m	54° 23' N 09° 32' E	I	L	C	EF184686	EF184819	EF184800
Sphagnum fimbriatum	344	Germany, Jagen	12 m	54° 21' N 10° 03' E	D	E	na	EF184690	EF184855	-
Sphagnum fimbriatum	350	UK, Cors Farlais, Carmarthenshire	300m	51° 54' N 04° 06' W	C	A	A	EF184719	EF184818	EF184801
Sphagnum fimbriatum	351	UK, Rhos Rydd, Cardiganshire	-	04° 44' N 52° 03' W	D	M	C	EF184688	EF184820	EF184802
Sphagnum fimbriatum	357	Sweden, Göteborg, Halland	10 m	57° 31' N 12° 10' E	D	E	C	EF184689	EF184855	EF184803
Sphagnum fimbriatum	366	France, Louargat	-	48° 35' N 03° 37' W	na	B	B	-	EF184893	EF184804
Sphagnum fimbriatum	367	France, Belle Isle-en-Terre	-	48° 28' N 03° 37' W	na	B	B	-	EF184848	EF184805
Sphagnum fimbriatum	368	France, Bretagne	-	48° 35' N 03° 37' W	na	B	B	-	EF184843	EF184806
Sphagnum fimbriatum	372	Estonia, Nätsi	-	58° 30' N 24° 04' E	D	E	H	EF184706	EF184842	EF184807
Sphagnum fimbriatum	373	Estonia, Tartu	-	58° 23' N 27° 06' E	D	D	C	EF184717	EF184840	EF184808
Sphagnum fimbriatum	374	Estonia, Tartu	-	58° 23' N 27° 06' E	D	D	C	EF184654	EF184844	EF184809
Sphagnum fimbriatum	375	Estonia, Rapla county	-	59° 02' N 24° 29' E	K	E	C	EF184705	EF184835	EF184810
Sphagnum fimbriatum	403	Britain, Berkshire	-	51° 13' N 01° 13' W	C	B	B	EF184651	EF184846	EF184811
Sphagnum fimbriatum	409	Belgium, Stekene	-	51° 11' N 03° 59' W	J	E	C	EF184718	EF184850	EF184812
Sphagnum fimbriatum	410	Belgium, Matagne la Grande	-	50° 06' N 04° 38' E	na	na	C	-	-	EF184813
Sphagnum fimbriatum	440	USA, Kansas	-	37° 26' N 97° 37' W	M	D	C	EF184723	EF184897	EF184814
Sphagnum fimbriatum	441	USA, Colorado	-	39° 33' N 105° 50' W	D	E	J	EF184722	EF184898	EF184815
Sphagnum fimbriatum	444	USA, Boothbay	30 m	43° 55' N 69° 38' W	D	E	K	EF184724	EF184895	EF184817
Sphagnum fimbriatum	446	USA, Maryland	-	49° 30' N 79° 18' W	T	E	C	EF184721	EF184899	EF184816

Species	Number of populations	Locality	m a.s.l	Longitude/Latitude	Haplotype					
					GapC	RAPDa	ITS	GapC	RAPDa	ITS
Sphagnum squarrosum	16	Norway, Sor-Trondelag	1 m	63° 42' N 08° 46' E	na	na	C	-	-	EF184949
Sphagnum squarrosum	17	Norway, Svalbard, Nordenskiöld Land	-	77° 55' N 14° 38' E	G	P	na	EF184924	EF185013	-
Sphagnum squarrosum	18	Norway, Sogn og Fjordane	345 m	62° 00' N 10° 00' E	C	C	B	EF184908	EF185054	EF184950
Sphagnum squarrosum	19	Norway, Aure	20 m	63° 15' N 08° 31' E	na	E	B	-	EF185041	EF184951
Sphagnum squarrosum	30	Pyrenees, Lac de Oredon	1900 m	42° 49' N 00° 10' E	C	B	B	EF184916	EF185055	EF184952
Sphagnum squarrosum	40	Switzerland, Eriz, Rotmoos	-	46° 54' N 07° 36' E	na	A	B	-	EF185014	EF184953
Sphagnum squarrosum	81.1	Austria, Lungau	1260 m	48° 06' N 12° 52' E	na	B	B	-		EF184954
Sphagnum squarrosum	85.1	Austria, Filzmoos	480 m	48° 06' N 12° 53' E	B	H	C	EF184946	EF185018	EF184955
Sphagnum squarrosum	96	Germany, Wasenmoos	460 m	47° 41' N 09° 35' E	H	J	B	EF184914	EF185021	EF184956
Sphagnum squarrosum	101	Germany, Hüven	8 m	52° 46' N 07° 34' E	B	Q	C	EF184900	EF185056	EF184957
Sphagnum squarrosum	103	Germany, Butterloch	200 m	51° 35' N 10° 18' E	B	F	C	EF184938	EF185028	EF184957
Sphagnum squarrosum	105	Bulgaria, Vitosha mountains	1750 m	40° 22' N 23° 22' E	I	na	B	EF184909	-	EF184958
Sphagnum squarrosum	107	Sweden, Mulön	-	65° 37' N 22° 17' E	B	N	na	EF184901	EF185023	-
Sphagnum squarrosum	112	Germany, Göttingen	5 m	51° 34' N 10° 08' E	B	H	C	EF184934	EF185022	EF184959
Sphagnum squarrosum	134	Russia, Moscow	-	55° 30' N 37° 30' E	B	H	C	EF184935	EF185045	EF184959
Sphagnum squarrosum	135	Latvia, locality 7.	-	56° 40' N 25° 55' E	D	J	C	EF184902	EF185046	EF184959
Sphagnum squarrosum	136	Latvia, locality 8.	-	56° 37' N 26° 20' E	B	I	C	EF184937	EF185025	EF184960
Sphagnum squarrosum	140	Latvia, locality 1.	-	56° 40' N 25° 55' E	D	I	C	EF184904	EF185058	EF184961
Sphagnum squarrosum	141	Latvia, locality 5.	-	56° 37' N 26° 20' E	C	C	B	EF184912	EF185012	EF184962
Sphagnum squarrosum	143	Estonia, Nigula	10 m	24° 40' N 58° 01' E	C	G	C	EF184915	EF185008	EF184963
Sphagnum squarrosum	147	Hungary, Velencei-tó	100 m	47° 12' N 18° 33' E	B	C	C	EF184936	EF185043	EF184964
Sphagnum squarrosum	151	Hungary, Köcse-tó	250 m	47° 01' N 16° 49' E	B	O	na	EF184929	EF185024	-
Sphagnum squarrosum	154	France, Walleis-Auemberg	-	50° 14' N 03° 41' E	B	M	C	EF184939	EF185020	EF184965
Sphagnum squarrosum	155	Finland, Hyrynsalmi	-	64° 19' N 29° 00' E	C	B	B	EF184926	EF185010	EF184966
Sphagnum squarrosum	156	Finland, Vörtsila	-	61° 56' N 29° 42' E	C	H	C	EF184913	EF185009	EF184967
Sphagnum squarrosum	157	Norway, Svalbard,Van Mijenfjorden	-	77° 49' N 16° 10' E	G	D	C	EF184923	EF185011	EF184968
Sphagnum squarrosum	158	Finland, Oulu	10 m	64° 58' N 25° 56' E	A	H	C	EF184940	EF185019	EF184969
Sphagnum squarrosum	159	Finland, Piipola	25 m	64° 22' N 25° 40' E	D	J	C	EF184903	EF185036	EF184970
Sphagnum squarrosum	160	Finland, Vieremä	-	63° 19' N 26° 32' E	B	B	B	EF184941	EF185035	EF184971
Sphagnum squarrosum	161	Finland, Hailuoto	10 m	65° 05' N 24° 46' E	G	R	C	EF184922	EF185027	EF184972
Sphagnum squarrosum	175	Slovakia, High Tatra mountains	1335 m	20° 03' N 49° 07' E	C	B	B	EF184910	EF185017	EF184973
Sphagnum squarrosum	176	Slovakia, High Tatra mountains	1080 m	49° 12' N 20° 16' E	C	B	na	EF184911	EF185042	-
Sphagnum squarrosum	180	Slovakia, Poprad Basin	680 m	49° 03' N 20° 17' E	na	B	B	-	EF185061	EF184974

Continued

Species	Number of populations	Locality	m a.s.l	Longitude/Latitude	Haplotype			GenBank accession number		
					GapC	RAPDa	ITS	GapC	RAPDa	ITS
Sphagnum squarrosum	239	Sweden, Långmossen	0 m	59˚ 58' N 17˚ 18' E	G	H	C	EF184948	EF185015	EF184975
Sphagnum squarrosum	240	Sweden, Norra Skrakskär (island)	3 m	59˚ 35' N 19° 20' E	na	I	C	-	EF185033	EF184976
Sphagnum squarrosum	241	Sweden, Rödskäret (island)	2 m	59˚ 36' N 19˚ 27' E	na	H	C	-	EF185034	EF184977
Sphagnum squarrosum	242	Sweden, Manskär (island)	2 m	59˚ 27' N 19˚ 31' E	na	na	C	-	-	EF184978
Sphagnum squarrosum	243	Sweden, Storön (island)	2 m	55° 11 N 20° 51' E	na	na	C	-	-	EF184979
Sphagnum squarrosum	264	Spain, locality 2005/9.	640 m	43° 02' N 02° 42' W	B	na	C	EF184947	-	EF184980
Sphagnum squarrosum	265	France, locality 2005/3.	1030 m	45° 57' N 03° 41' E	C	A	B	EF184907	EF185052	EF184981
Sphagnum squarrosum	289	Austria, Seemoos	1670 m	47° 10' N 13° 47' E	C	C	B	EF184928	EF185039	EF184982
Sphagnum squarrosum	292	Romania, Kelemen havasok	800 m	47° 14' N 25° 16' E	C	C	B	EF184917	EF185059	EF184983
Sphagnum squarrosum	321	Poland, Oswiecim basin	-	50° 01' N 19° 06' E	na	na	C	-	-	EF184984
Sphagnum squarrosum	322	Hungary, Kismohos	300 m	48° 15' N 20° 13' E	F	M	C	EF184942	EF185031	EF184985
Sphagnum squarrosum	323	Hungary, Tegda	440 m	48° 25' N 21° 29' E	B	I	C	EF184931	EF185032	EF184986
Sphagnum squarrosum	324	Poland, Silesian upland	740 m	51° 11' N 18° 38' E	na	na	B	-	-	EF184987
Sphagnum squarrosum	325	Poland, Maly Mts.	-	50° 51' N 14° 26' E	na	na	B	-	-	EF184988
Sphagnum squarrosum	326	Poland, Rybnicki Plateau	-	50° 08' N 18° 27' E	na	na	B	-	-	EF184989
Sphagnum squarrosum	333.1	Montenegro, Durmitor	1320 m	42° 83' N 18° 23' E	C	C	na	EF184927	EF185038	-
Sphagnum squarrosum	333.2	Montenegro, Durmitor	1320 m	42° 83' N 18° 23' E	C	C	B	EF184921	EF185040	EF184990
Sphagnum squarrosum	334	Serbia, Golija 03/105/6	1480 m	43° 20' N 20° 15' E	C	H	B	EF184944	EF185015	EF184991
Sphagnum squarrosum	335	Serbia, Golija	1480 m	43° 20' N 20° 15' E	na	B	B	EF184920	EF185060	EF184992
Sphagnum squarrosum	339	Norway, Troms	0 m	69° 47' N 21° 02' E	G	K	C	EF184925	EF185057	EF184993
Sphagnum squarrosum	341	Russia, Rybachy	2 m	55° 11' N 20° 51' E	na	na	C	-	-	EF184994
Sphagnum squarrosum	344	Germany, Jagen	12 m	54° 23' N 09° 31' E	A	H	C	EF184940	EF185030	EF184995
Sphagnum squarrosum	354	Russia, Bolshojslovetski Island	-	64° 42' N 39° 44' E	D	T	C	EF184905	EF185026	EF184996
Sphagnum squarrosum	355	Russia, Arckhangelsk region	-	64° 42' N 39° 44' E	D	S	C	EF184906	EF185029	EF184997
Sphagnum squarrosum	356	Russia, Kola Peninsula	-	68° 05' N 39° 50' E	B	G	C	EF184933	EF185053	EF184998
Sphagnum squarrosum	360	Göteborg, Rammsjödal	10 m	57° 31' N 12° 10' E	B	E	D	EF184932	EF185037	EF184999
Sphagnum squarrosum	364	France, Spezet	-	48° 11' N 03° 42' W	na	na	C	-	-	EF185000
Sphagnum squarrosum	370	Estonia, Nigula	-	58° 23' N 27° 06' E	H	B	A	EF184919	EF185016	EF185001
Sphagnum squarrosum	371	Estonia, Tartu	-	58° 23' N 27° 06' E	B	L	C	EF184930	EF185044	EF185002
Sphagnum squarrosum	383	Slovenia, Šijec	1200 m	46° 20' N 13° 59' E	J	C	B	EF184918	EF185049	EF185003
Sphagnum squarrosum	393	Finland, Kuopio	-	62° 52' N 27° 41' E	K	B	B	EF184945	EF185050	EF185004
Sphagnum squarrosum	394	Finland, Liminka	-	64° 49' N 25° 23' E	E	E	C	EF184943	EF185048	EF185005
Sphagnum squarrosum	413	Belgium, Lux, Bellevaux	-	49° 50' N 05° 13' E	na	A	B	-	EF185051	EF185006
Sphagnum squarrosum	414	Belgium, Slekene		51° 11' N 03° 59' W	na	F	C	-	EF185047	EF185007
