# Supplementary material for: Multilocus dataset reveals demographic histories of two peat mosses in Europe
Source: BMC Evol Biol. 2007 Aug 22;7:144. doi: 10.1186/1471-2148-7-144 (PMC2018716; doi:10.1186/1471-2148-7-144)
Supplement: Additional file 2 — Primers used in this study. Names of newly designed primers are in bold. 1st and 2nd refer to the first and the second round of a semi nested PCR. In the second round PCR 1 μl 10 × diluted first round product was used as template. [file 1471-2148-7-144-S2.pdf]

Additional file 2 Usage and sequences of primers used in this study. Names of newly designed primers are in bold. 1st and 2nd refer to the first and the second round of a semi nested PCR. In the second round PCR 1 µl 10x diluted first round product was used as template.

Region	Name	Usage	Sequence (5`-3`)	Design based on	Annealing T and length of extension
ITS	ITS1	amplification	TCCGTAGGTGAACCTGCGG	White et al. 1990	1st 50 °C, 60 s
	ITS4	amplification and sequencing	TCCTTCCGCTTATTGATATGC	Baum et al. 1998	2nd 54 °C, 60 s
	**ITS1-int.**	amplification and sequencing	CACACAGAGCGGTAAACCCTGC	*Sphagnum* sequences	
RAPDa	A-F	amplification	AACCAAGTGAATTTGGAATGC	Shaw et al. (2003a)	1st 56 °C, 90 s
	A-R	amplification and sequencing	AGGAGCGGAAGGCAAATG	Shaw et al. (2003a)	2nd 57 °C, 90 s
	**RAPDa-forw.**	amplification and sequencing	GATCCAGCCAAATCCACAAGATTCA	*Sphagnum* sequences	
	**RAPDa-rev.**	sequencing	CCTTYGACAAGGTTCGTGKTCTACTC	*Sphagnum* sequences	
	**RAPDa-forw.int.**	sequencing	TCCTCGATCCAGBAGATGGTAGA	*Sphagnum* sequences	
	AiR	sequencing	CAGAATGGCGAGCTTCCT	Shaw et al. (2003a)	
	AiF	sequencing	CAGCATTTTGGCTTTCCAAG	Shaw et al. (2003a)	

Baum DA, Small RL, Wendel JF: **Biogeography and floral evolution of baobabs (*Adansonia*, Bombacaceae) as inferred from multiple data sets.** *Syst. Biol.* 1998, 47: 181-207

White TJ, Burns T, Lee S, Taylor J: **Amplification and direct sequencing of fungal ribosomal RNA genes for phylogenetics.** In: PCR protocols, *a Guide to Methods and Applications*. Edited by Innis M, Gelfand D, Sninsky J, White T. San Diego, California, Academic Press 1990, 315-322
